# Recombinant human secretory leukocyte protease inhibitor (rhSLPI) coated titanium enhanced human osteoblast adhesion and differentiation

**DOI:** 10.1038/s41598-023-50565-8

**Published:** 2023-12-27

**Authors:** Radchanon Leelasukseree, Wannapat Chouyratchakarn, Chayanisa Phutiyothin, Faprathan Pikwong, Onnicha Srisopar, Phornsawat Baipaywad, Suruk Udomsom, Podsawee Mongkolpathumrat, Chayarop Supanchart, Sarawut Kumphune

**Affiliations:** 1 Biomedical Engineering and Innovation Research Center, Chiang Mai University, Mueang Chiang Mai District, Chiang Mai, 50200 Thailand; 2 Biomedical Engineering Institute (BMEI), Chiang Mai University, Mueang Chiang Mai District, Chiang Mai, 50200 Thailand; 3 Cardio-Thoracic Technology Program, Chulabhorn International College of Medicine (CICM), Cooperative Learning Center, Thammasat University (Rangsit Center), Piyachart 2, 99 Moo 18 Klong Luang, Rangsit, Pathumthani 12120 Thailand; 4 Department of Oral and Maxillofacial Surgery, Faculty of Dentistry, Chiang Mai University, Chiang Mai, 50200 Thailand

**Keywords:** Cell biology, Health care, Materials science

## Abstract

Osseointegration is vital to success in orthopedic and dental reconstructions with implanted materials. The bone matrix or cells—particularly osteoblasts—are required to achieve functional contact on the implant surface. Osteoblast induction is therefore essential for osteogenesis to occur. Enhancement of osteoblast adhesion, proliferation, and differentiation, particularly by implant surface modifications, have been found challenging to develop. Secretory Leukocyte Protease Inhibitor (SLPI), a cation ionic protein with anti-inflammatory and anti-bacterial activities, showed activation in osteoblast proliferation and differentiation. However, the effects of coating recombinant human (rh) SLPI on a titanium alloy surface on human osteoblast adhesion, proliferation, and differentiation has never been investigated. In this study, titanium alloys (Ti–6Al–4V) were coated with rhSLPI, while human osteoblast adhesion, proliferation, differentiation, actin cytoskeletal organization, and gene expressions involved in cell adhesion and differentiation were investigated. The results indicate that coating titanium with 10–100 µg/ml rhSLPI enhanced the physical properties of the Ti surface and enhanced human osteoblast (hFOB 1.19) cell adhesion, activated actin dynamic, enhanced adhesive forces, upregulated *integrins α1*, *α2*, and *α5*, enhanced cell proliferation, mineralization, alkaline phosphatase activity, and upregulated *ALP*, *OCN*, and *Runx2*. This is the first study to demonstrate that coating SLPI on titanium surfaces enhances osseointegration and could be a candidate molecule for surface modification in medical implants.

## Introduction

The revolutionary clinical therapeutic technique of osseointegration has been used in dentistry and orthopedics for several decades. Restoration of the edentulous space in dental implant surgery or amputation surgery is achieved by forming connections between the bone matrix and the metal surface of the prosthetic metal implant^1,2^. Titanium alloys and other metal surfaces are widely employed due to their chemical and structural malleability, as well as their physical qualities and biocompatibility^3^. As of 2022, the global population is expected to become increasingly overweight and elderly, both of which make individuals more vulnerable to fractures^4^ and tooth loss^5^. The efficacy of osseointegration has been demonstrated in clinical settings, yet bone remodeling imbalances brought on by diseases including diabetes mellitus, osteoporosis, and periodontitis can result in dental implant failure among such individuals^6,7^. These pathological conditions could influence the failure of artificial limb transplants^8^. Hence, further research into strengthening osseointegration is required to enhance the quality of life for people with health complications.

Secretory Leukocyte Protease Inhibitor (SLPI) is a ~ 12 kDa cationic protein that plays a role in antagonizing inflammatory responses and upregulating tissue regeneration^9^. SLPI induces anti-inflammatory responses in odontoblasts^10^ and also mediates the parathyroid hormone (PTH) pathway by upregulating the expression of SLPI to cause differentiation and mineralization of osteoblasts. Consequently, osteoclast activity decreases, and new bone production is induced^11^. SLPI, therefore, has therapeutic potential by providing regenerative responses via cell proliferation and differentiation. Additionally, a previous study reported that the injection of SLPI in MC3T3-E1 cells—a preosteoblastic cell line from mouse calvaria—increases the cells’ survival and adhesion to the titanium surface^12^. This suggests that SLPI may promote differentiation and mineralization of MC3T3-E1 preosteoblasts. Choi et al.^13^ further reported and confirmed that SLPI could upregulate genes involved in osteoblast differentiation in MC3T3-E1 cells. Nonetheless, previous studies faced several limitations. First, the effects of SLPI on human osteoblasts adhesion, proliferation, differentiation as well as molecular insights have never been investigated. Moreover, pre-treatment of recombinant human SLPI protein on osteoblasts seems impractical and not relevant in a clinical context. Therefore, coating SLPI on the titanium alloy surface is more practical for potential clinical applications in the near future. Thus, the purpose of this research is to disseminate information about the potential clinical application of coated recombinant human secretory leukocyte protease inhibitor (rhSLPI) on titanium to improve osteoblast adhesion, proliferation, and differentiation.

## Results

### Surface characterization of Ti-coated by rhSLPI

**Figure 1:**
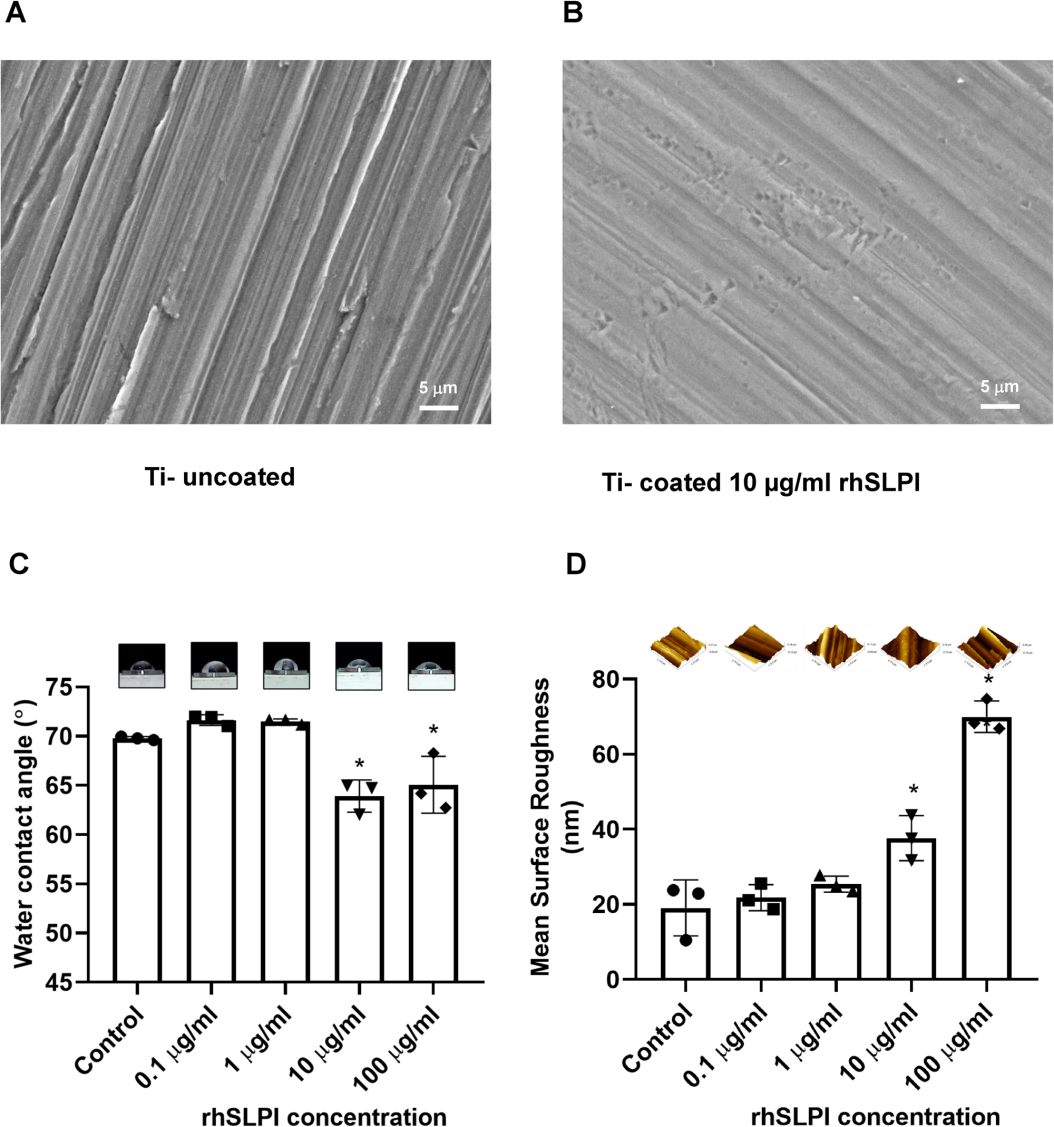
Surface characterization of Ti-coated by rhSLPI. The scanning electron microscope of the surface of (**A**) Ti surface and (**B**) rhSLPI-coated Ti, (**C**) the effect of rhSLPI coating on hydrophobicity property by contact angle analysis and (**D**) the surface roughness by AFM.

The surface morphology of coated and non-coated titanium discs with rhSLPI was showed in Fig. 1a, b. The untreated surface of titanium was observed to have clear and deep lines, which attributed to the cutting process, while the treated surface of titanium showed a patina-like appearance, which caused the lines to become blurred and shallow. Coating of rhSLPI on Ti, particularly 10, and 100 µg/ml, significantly reduced hydrophobicity by reducing the contact angle value (Fig. 1c) and enhanced the surface roughness (Fig. 1d).

### Coated rhSLPI on Ti surface enhanced osteoblast adhesion without cellular toxicity

**Figure 2:**
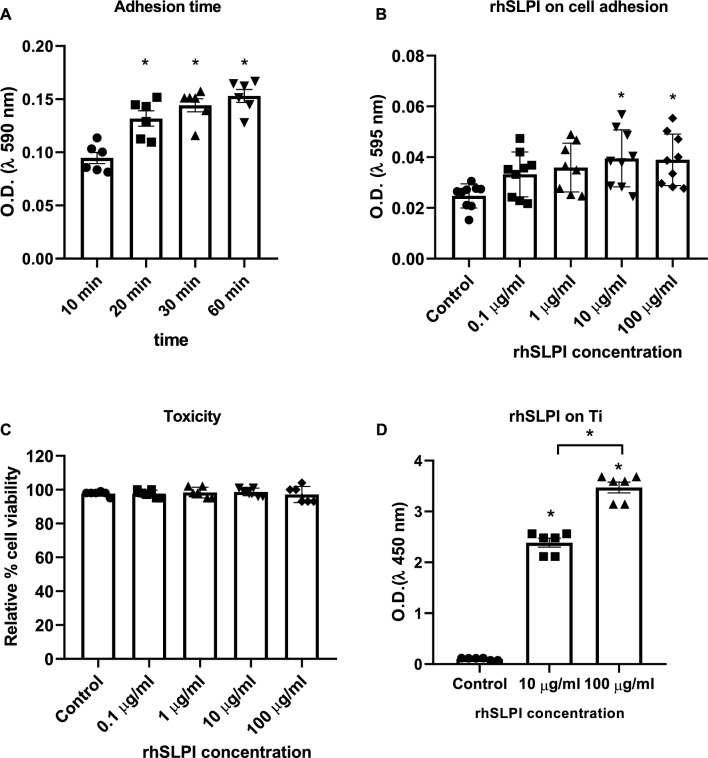
Optimization of timing of human osteoblast (hFOB 1.19) adhesion on (**A**) Ti surface, (**B**) effect of varied coated concentrations of rhSLPI on Ti through 20 min of adhesion on the number of hFOB 1.19 adherent cells, (**C**) the cellular toxicity of varied coated concentrations of rhSLPI, and (**D**) determination of rhSLPI found on the Ti surface by ELISA. *p < 0.05 vs control (ANOVA) or as indicated.

To determine the effect of rhSLPI coating on a Ti surface to enhance osteoblast adhesion, the timing of human osteoblasts (hFOB 1.19) adhesion on the Ti surface was first optimized. The results show that incubation of hFOB 1.19 cells for 20 min after seeding gave a significant number of attached cells when compared to a 10-min incubation period. Longer periods of incubation for 30 and 60 min did not show significant differences when compared to the 20-min period (Fig. 2a).

Several concentrations of rhSLPI, including 0.1, 1, 10, and 100 µg/ml were then coated on the Ti surface for 24 h, which determined the adhesion of hFOB 1.19 cells. The results indicate that coating the Ti surface with 10 and 100 µg/ml rhSLPI significantly enhanced hFOB 1.19 adhesion (Fig. 2b). These concentrations of rhSLPI showed no cellular toxicity on hFOB 1.19 cells (Fig. 2c).

To ensure that the rhSLPI was successfully coated on the Ti surface, enzyme-linked immunosorbent assay (ELISA) was performed. The results confirmed the presence of rhSLPI on the Ti surface in a dose-dependent manner (Fig. 2d).

### rhSLPI coating enhanced osteoblast cell spreading on Ti surface

**Figure 3:**
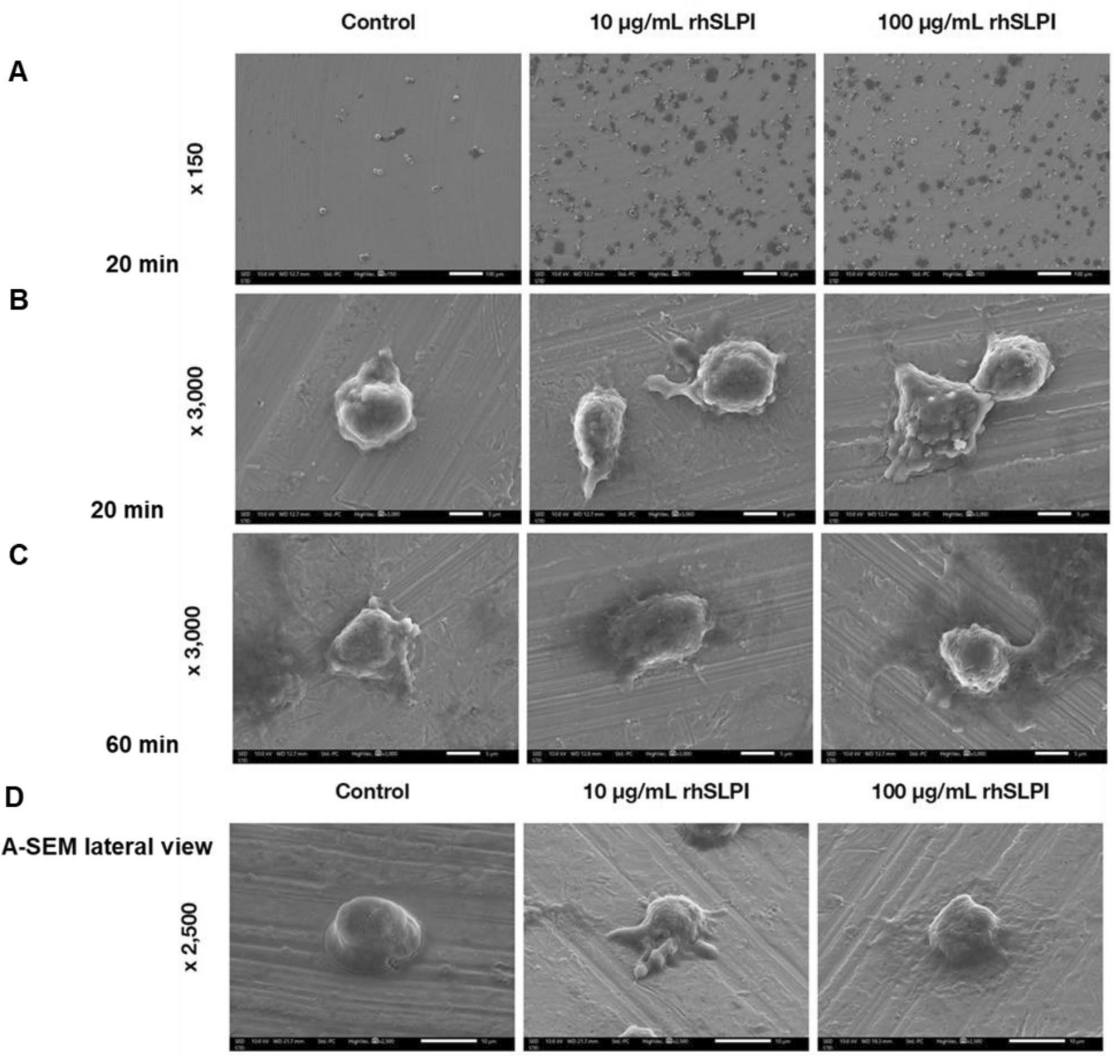
Scanning Electron Microscope of hFOB 1.19 shows cellular morphological appearances of adhered osteoblasts on rhSLPI-coated Ti.

After seeding hFOB 1.19 on the Ti disc and incubating each sample separately for 20 and 60 min, the morphological appearances of the adhered hFOB 1.19 cells were observed by scanning electron microscopy (SEM) (Fig. 3). The hFOB 1.19 cells in the controlled, uncoated Ti surface, had a small size and a round shape. In contrast, hFOB 1.19 cells attached on the Ti surface coated with 10 µg/ml rhSLPI increased in cell size with parts along the edges of the cell extended and stretched out. Moreover, coating the Ti surface with 100 µg/ml rhSLPI enhanced cell size, spreading, and expansion, resulting in the enlargement of the cell’s contact area. The expansion of cell morphology could be clearly observed when cells were incubated for 60 min.

### rhSLPI coating enhanced adhesive forces of osteoblast on Ti surface

**Figure 4:**
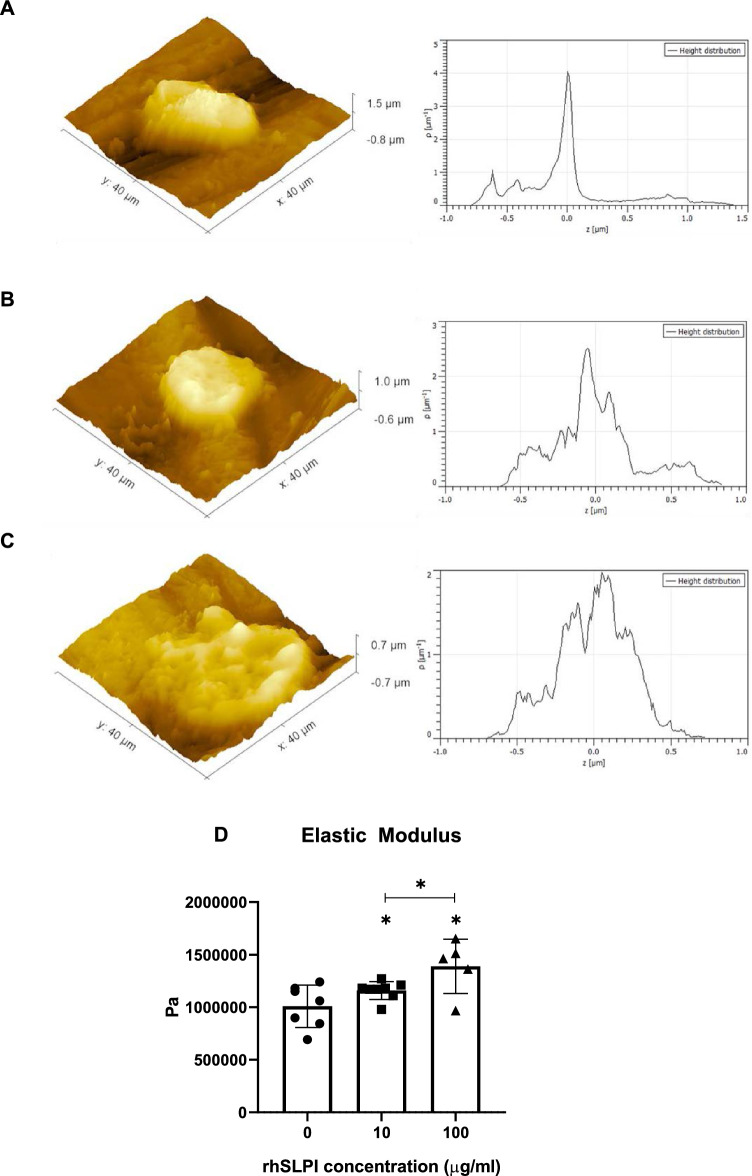
Atomic force microscopy (AFM) images of (**A**) hFOB 1.19 cell on non-coated Ti, (**B**) on 10 µg/ml rhSLPI-coated Ti, and (**C**) on 100 µg/ml rhSLPI-coated Ti, (**D**) quantitative analysis of elastic modulus. * p < 0.05 vs control (ANOVA) or as indicated.

The atomic force microscopy (AFM) results show attached hFOB 1.19 cells displaying characteristics of longer cell diameter and lower altitude on Ti surface coated with 10 and 100 µg/ml of rhSLPI compared to the control with non-coated rhSLPI. This suggests that the edges of the cell were extended and stretched out (Fig. 4a–c).

The elastic modulus significantly increased in attached hFOB 1.19 cells on the Ti surface coated with 10 and 100 µg/ml of rhSLPI compared to the control (1.158696 ± 0.085056 MPa, 1.389039 ± 0.258967 MPa, and 1.009485 ± 0.201693 MPa, respectively p < 0.05, ANOVA) (Fig. 4d). These findings suggest that osteoblasts are more adhesive when seeded on Ti surfaces coated with rhSLPI.

### rhSLPI coating enhanced actin cytoskeleton accumulation along the cell edge

**Figure 5:**
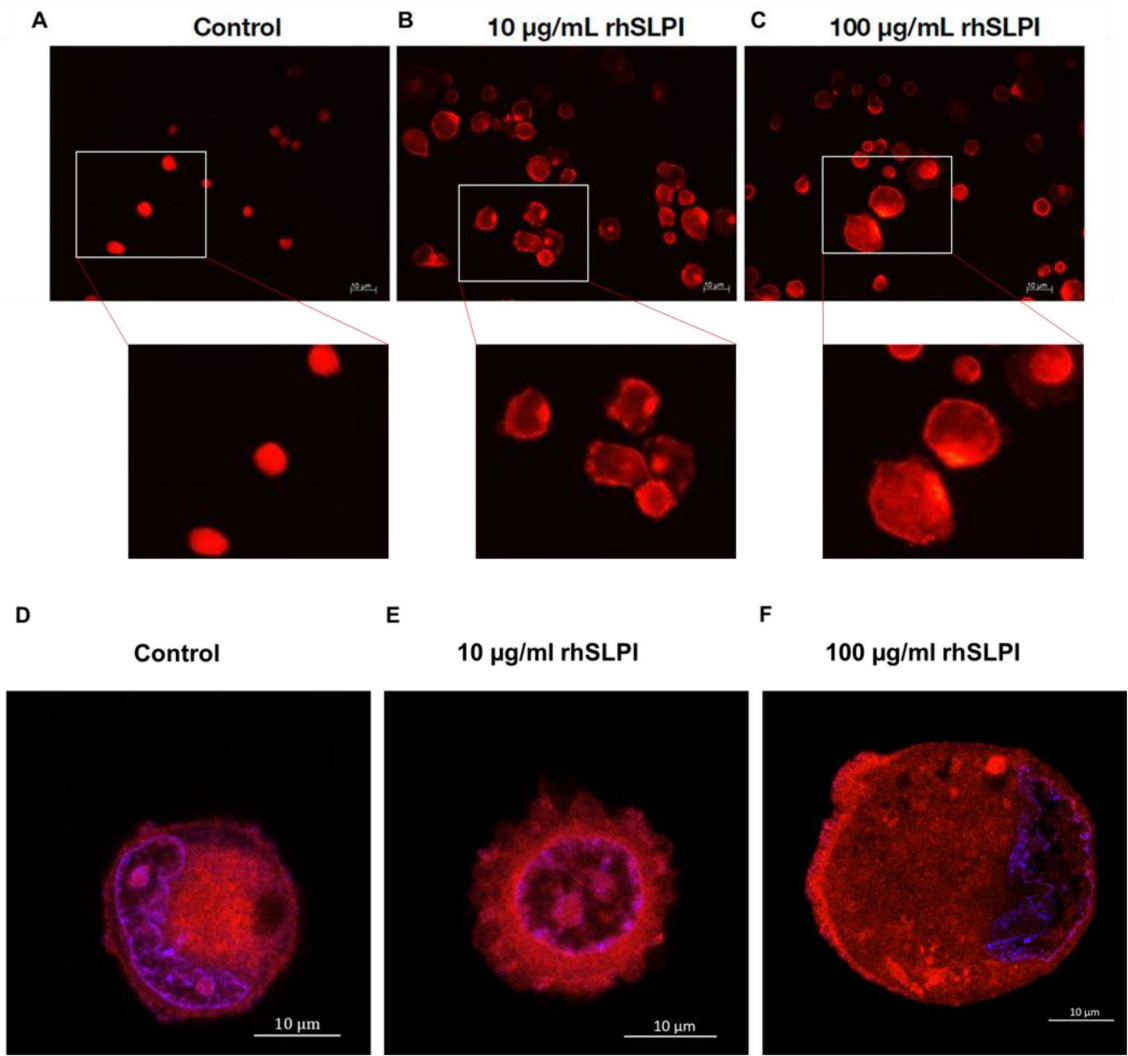
Fluorescence microscopy images of hFOB 1.19 cells on (**A**) Ti, (**B**) Ti coated with 10 µg/ml rhSLPI, and (**C**) Ti coated with 100 µg/ml rhSLPI were stained with phalloidin-TRITC (red). Confocal microscopy images of hFOB 1.19 cells on (**D**) Ti, (**E**) Ti coated with 10 µg/ml rhSLPI, and (**F**) Ti coated with 100 µg/ml rhSLPI were stained with phalloidin-TRITC (red) and counterstained with DAPI (blue) solution; fine details on the formation of the actin cytoskeleton (red) and the nucleus (blue) are shown.

The relationship between the effects of cell-substrate contact on cytoskeleton orientation, focal adhesion formation, and the shape and adhering nature of cells in response to certain extracellular environments has been discussed^14^. In this study, the effects of Ti surface coating by rhSLPI on cytoskeleton orientation and focal adhesion formation were observed by F-actin staining with phalloidin-conjugated TRITC. The distribution of actin filaments was shown to be largely concentrated at the peripheries of the attached hFOB 1.19 cells on Ti surfaces coated with 10 and 100 µg/ml rhSLPI for 20 min (Fig. 5b, c), when compared to control group that the actin homogenously accumulates inside the cell (Fig. 5a). Furthermore, the confocal images showed actin cytoskeleton accumulation at the peripheries of the cell that seeded on rhSLPI coated Ti (Fig. 5e, f) but not in control group (Fig. 5d), indicating the contact locations of the cell-substrate interaction.

### The rhSLPI coating enhanced gene expression of osteoblast adhesion molecules

**Figure 6:**
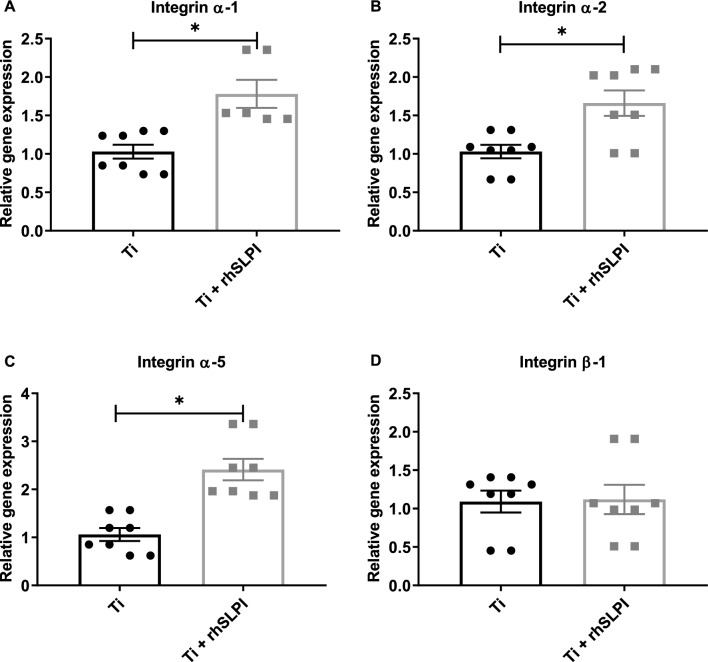
Quantitative RT-PCR results in assessing levels of mRNA expression in osteoblast adhesion molecules, integrin subunits (**A**) α1, (**B**) α2, (**C**) α5, and (**D**) β1. *p < 0.05 vs Ti (unpaired-t test).

Upon having cell-substate interactions, osteoblasts express numerous cell adhesion molecules such as integrins. The surface chemical composition influences the formation and expression of integrin subunits in osteoblasts grown on Ti. The results show that coating the Ti surface with 10 µg/ml rhSLPI could significantly enhance the expression of integrin subunits α1, α2, and α5, but not β1 (Fig. 6).

### rhSLPI coating enhanced osteoblast proliferation

**Figure 7:**
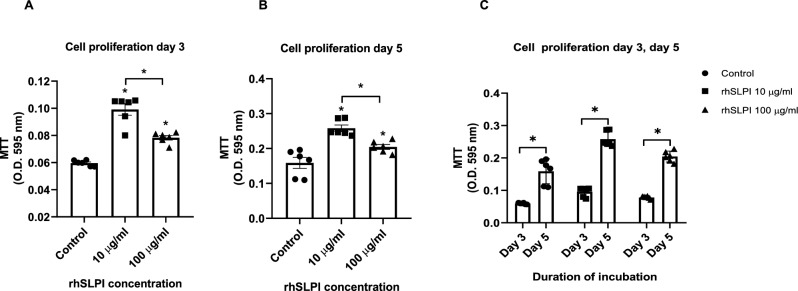
Effect of rhSLPI surface coating on Ti surface on cell proliferation at (**a**) Day 3, (**b**) Day 5, and (**c**) comparison between Day 3 and Day 5. *p < 0.05 vs control (ANOVA) or as indicated.

The effect of rhSLPI surface coating on hFOB 1.19 cell proliferation was performed on Days 3 and 5. The results showed that coating the Ti surface with 10 µg/ml rhSLPI could significantly enhance hFOB 1.19 cell proliferation when compared to the non-coated control group (Fig. 7). However, there was no significant increase in hFOB 1.19 cell proliferation on Ti surfaces coated with 100 µg/ml rhSLPI.

### rhSLPI coating enhanced alkaline phosphatase activity and mineralization

**Figure 8:**
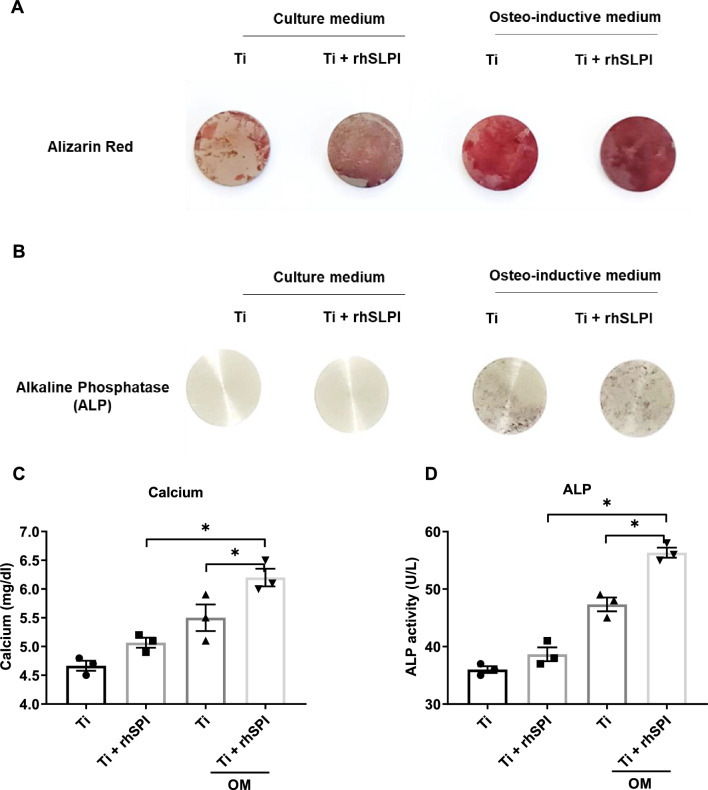
Effect of rhSLPI surface coating on Ti surface on (**A**) mineralization for 14 days by Alizarin Red S staining. (**B**) Alkaline phosphatase activity. The (**C**) calcium concentration and (**D**) ALP activity in culture medium was also determined by biochemical analyzer.

Mineralization of hFOB 1.19 cells was assessed by Alizarin Red S staining. The findings show that there was an increase in the level of mineral deposition on the Ti surface coated with 10 µg/ml rhSLPI compared with the non-coated Ti surface, in both non-induced and differentiation induction groups (Fig. 8a). The calcium concentration in culture medium was increased in condition of Ti surface coated with 10 µg/ml rhSLPI compared with the non-coated Ti surface, in both non-induced and differentiation induction groups (Fig. 8c).

The alkaline phosphatase (ALP) activity was also assessed by staining the ALP activity to hydrolyze the phosphate group on the substrate, 5-Bromo-4-chloro-3-indolyl phosphate (BCIP) to form a blue colored intermediate. The results showed that there was an increase in the level of ALP activity on the Ti surface coated with 10 µg/ml rhSLPI compared with the non-coated Ti surface, in both non-induced and differentiation induction groups (Fig. 8b). The ALP activity in culture medium was increased in condition of Ti surface coated with 10 µg/ml rhSLPI compared with the non-coated Ti surface, in both non-induced and differentiation induction groups (Fig. 8d).

### rhSLPI coating enhanced gene expression of osteoblast differentiation markers

**Figure 9:**
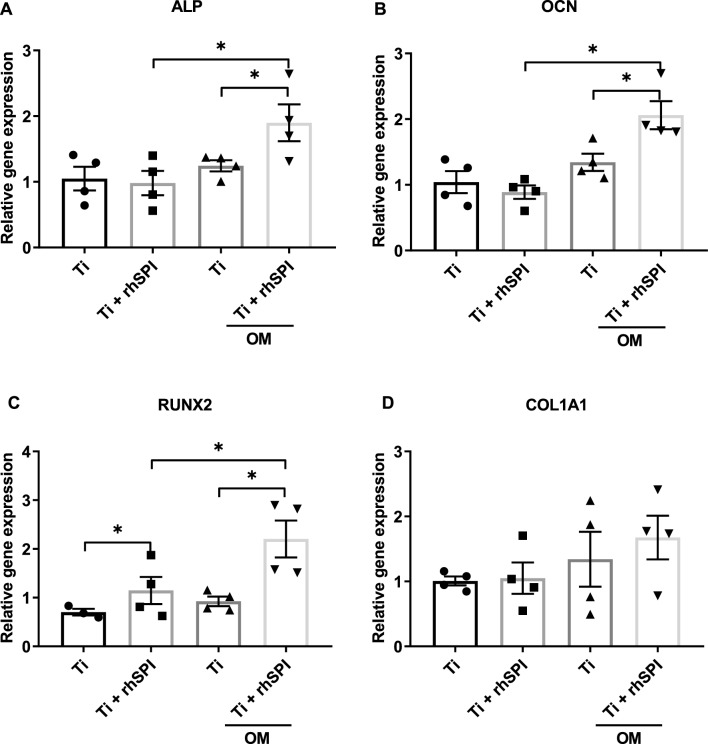
Quantitative RT-PCR results in assessing levels of mRNA expression in osteoblast differentiation markers, (**A**) ALP, (**B**) OCN, (**C**) Runx2, and (**D**) COL1A1. *p < 0.05 vs control (ANOVA) or as indicated.

Osteoblast differentiation was evaluated by determining levels of gene expression of differentiation markers, including alkaline phosphatase (ALP), osteocalcin (OCN), runt-related transcription factor 2 (Runx2), and collagen type I alpha 1 (COL1A1). The results reveal that there was an increase in the expression levels of ALP, OCN, and Runx2 (Fig. 9a–c) but not COL1A1 (Fig. 9d) in osteoblasts that were cultured in an osteogenic inductive condition on Ti coated with 10 µg/ml rhSLPI. In contrast, osteoblasts cultured in the non-inductive condition were shown to have a significant increase only in the expression of the Runx2 gene upon coating Ti with 10 µg/ml rhSLPI (Fig. 9c).

## Discussion

In the fields of dentistry and orthopedics, osseointegration is well known for its therapeutic benefits to restore cellular functions in damaged bone cells. Through performing dental implant and amputation surgery, the bone matrix can form connections with the metal surface of the prosthetic metal implant to regenerate bone cells^1,2^. However, these implant and healing responses tend to be unsuccessful in individuals with metabolic illnesses such as diabetes mellitus, osteoporosis, and periodontitis due to insufficient bone regeneration and bone degradation^6,7^. Therefore, a different clinical approach is required to improve implant conditions in patients with chronic illnesses.

After implantation, there are several biological responses orchestrated in the process. This includes early anti-inflammatory responses, angiogenesis, and osteogenesis^15^. During the early anti-inflammatory period, macrophages and other immune cells enter the peri-implant gap and secrete cytokines, which will adhere to the implant surface^16,17^, forming the peri-implant mucosa, a soft tissue around the implant^18^. Successes in osseointegration for dental implantation are strongly dependent upon transmucosal healing and the stability of peri-implant mucosa^17^. Because of the difference in the direction of gingival fibers in dental implants, bacteria can penetrate through the epithelial cell layer and other connective tissues causing the peri-implant mucosa to break down^17^. Thus, maintaining and improving the health of peri-implant mucosa and upregulating anti-inflammatory responses are crucial for implantation. Subsequently, new blood vessels were formed as a result of angiogenesis, followed by the migration of mesenchymal stem cells^18,19^. As mesenchymal stem cells differentiate into osteocytes, mature lamellar bone was formed and the peri-implant interface was replaced, securing the stability of the implant^17^. These reports highlight the role of biological responses in implantation. Therefore, methods that further upregulate anti-inflammatory responses or inhibit inflammation and enhance osteogenesis are vital for reducing implant failures in ill and elderly patients.

Titanium alloys are most commonly used as materials for prosthetic implants^3^. However, the roughness of the implant surface promotes the adhesion of bacteria on the titanium surface, causing prolonged inflammation and leading to implant disintegration^20^. Thus, synthetic surface modifications such as a zirconia coating, which involves the use of silica, magnesium, nitrogen, carbon, hydroxyapatite, calcium phosphate, dopamine, and graphene, can be implemented to mitigate the effects of bacterial infections as well as stimulate bone cell adherence^21^. Despite these positive effects of synthetic surface modifications, other factors that cause a reduction in cell inflammation, an enhancement in wound healing, and stimulation of bone remodeling processes have been overlooked. Therefore, a novel therapeutic target is needed to further improve implant conditions during osseointegration.

Secretory leukocyte protease inhibitor (SLPI) is an immune-associated protein that plays a role in anti-inflammatory and anti-microbial responses mainly in epithelial cells of reproductive tracts, submucosal glands, and mucous linings in the respiratory tract^9,22^. SLPI mediates cell growth and prevents apoptosis from occurring by providing protection from secreted proteolytic enzymes^9^. Because of this, anti-inflammatory, anti-bacterial, anti-viral, and anti-apoptotic effects are upregulated, resulting in cell proliferation and differentiation^9^. A study conducted by Jeong et al. demonstrated that the pre-treatment of SLPI on mouse calvaria osteoblasts could enhance the adhesion of migration rate^12^. Furthermore, Choi et al. also showed how the pre-treatment of SLPI on mouse osteoblasts promotes differentiation and mineralization, suggesting the possible therapeutic potential of SLPI in improving success rates during osseointegration^13^. However, these studies were performed using non-human cells, and using pre-treatment strategies of SLPI does not represent real clinical applications in humans. Therefore, the current study aims to use a more clinically relevant approach to investigate the effects of coating SLPI on titanium to enhance human osteoblasts adhesion, proliferation, and differentiation.

Coating of rhSLPI on Ti seems to enhance the osteoblast cell adhesion due to the physical modification of the Ti surface. rhSLPI-coated Ti showed an increase in surface roughness and less hydrophobicity. The findings of the study indicate that concentrations of 10 and 100 µg/ml of rhSLPI demonstrate reduced hydrophobicity, as seen by a smaller contact angle, in comparison to concentrations of 0, 0.1, and 1 µg/ml of rhSLPI. The observed result may be elucidated by the existing literature on the conformational stability of SLPI. The structural configuration of the SLPI protein has been seen to have a resemblance to a boomerang, wherein the N-terminal and C-terminal domains constitute the two ends of the protein. In general, the structure of SLPI lacks a hydrophobic core and has little secondary structure characterized by hydrogen bonding^23^. For cellular and molecular explanation for the effect of rhSLPI to enhance osteoblast adhesion in this study highlighted on the rapid organization of the actin cytoskeleton was observed through the formation of actin filaments along the peripheries of the osteoblast to enhance expressions of osteoblast adhesion molecules: integrin subunits α1, α2, and α5 increased substantially, enhance cell proliferation upon adhering to the Ti surface, and upregulate genes involved in osteoblast differentiation by increasing in calcium level and alkaline phosphatase activity. The present work also indicates the potential of rhSLPI to induce osteogenic differentiation, as evidenced by its ability to enhance the expression of osteogenic differentiation marker genes when grown in an osteogenic induction medium. The osteogenic induction medium, comprising dexamethasone, ascorbic acid, and sodium β-glycerophosphate, has been demonstrated to stimulate the expression of osteogenic differentiation marker genes such as ALP, RUNX2, OCN, COLA1A, and etc.^24^. Previous research has indicated that exposing stem cells to this medium for a duration of 14–21 days can effectively augment the expression of these genes. In this work, it was shown that culturing the osteoblasts in osteogenic induction media for a duration of 14 days resulted in a modest increase in the expression of osteogenic differentiation marker genes, as compared to culturing them in a basic culture medium. Thus, the study presents strong evidence of the potential therapeutic improvement of osseointegration using coated rhSLPI on a titanium surface as SLPI regulates the formation and restoration of the bone matrix.

The present investigation posits that the application of rhSLPI at concentrations of 10 and 100 µg/ml on the surface of titanium (Ti) yields improved physical characteristics of the Ti surface. Additionally, this coating has the potential to augment the adhesion, proliferation, and differentiation of osteoblasts. The observed effects appear to exhibit a dose-dependent relationship, with the exception of osteoblast growth. The application of rhSLPI at concentrations of 10 and 100 µg/ml on the surface of titanium (Ti) resulted in a notable enhancement in cell proliferation compared to the control group that did not receive any treatment. However, it was observed that the concentration of 100 µg/ml of rhSLPI had a comparatively weaker impact on cell proliferation over both the 3-day and 5-day experimental durations. Based on the findings of the previous study, which indicated that SLPI has the ability to activate FAK and ERK signaling pathways^12^, resulting in an augmentation of cell proliferation, it can be hypothesized that administering a larger concentration of rhSLPI would further amplify the proliferative impact. Nevertheless, the cultivation of osteoblasts in 96-well plates for a duration of 3–5 days, accompanied by significant stimulation by rhSLPI, may result in excessive cell growth, thus influencing the accurate assessment of cell proliferation using the MTT test. The potential impact of cell detachment, particularly in overgrown populations, may be influenced by the iterative procedure of discarding the culture media and substituting it with chemicals. The potential outcome of this might be a decrease in the absorbance value of the formazan in the MTT experiment.

Nonetheless, several issues or limitations with the present study are yet to be considered. Although this was the first study to use a human-fetal osteoblastic cell model, it is not a suitable model for clinical use. The process of osseointegration tends to be performed on elderly patients, so an in vitro adult human osteoblastic cell model is more appropriate. Clinical specimens of adult human osteoblasts could be isolated and tested to yield more reliable results to determine the effectiveness of rhSLPI as a possible therapeutic candidate for osseointegration. Moreover, osseointegrated implants and amputation surgery tend to fail in patients with osteoporosis, periodontitis, and diabetes mellitus. However, the study was conducted under ideal conditions, so non-ideal conditions, such as environments that mimic osteoporosis and diabetes mellitus, were not tested. Therefore, it is unknown whether rhSLPI is a novel treatment for patients with chronic illnesses. To gather more data, culturing adult human osteoblasts in different environments with D-galactose and D-glucose will mimic non-ideal conditions of osteoporosis and diabetes mellitus, respectively. Additionally, the stability of the rhSLPI surface coating on the titanium surface was not tested in this study. In a clinical setting, coated titanium screws are packaged and exported to be used on patients. Because of this, it is yet to be determined whether rhSLPI loses its molecular function over time, so stability assays should be performed in future studies. Furthermore, although the study presents strong findings of rhSLPI’s role in increasing cell proliferation and differentiation, it is unclear whether coated rhSLPI could induce the formation of bone sarcoma. Therefore, performing immunohistochemistry (IHC) on retrieved tissue sections from an in vivo model could be used to determine the presence of bone sarcoma. Ultimately, findings from the present study strongly emphasize the necessity of performing transplants of coated rhSLPI titanium screws through an in vivo model since future studies could provide crucial pieces of evidence before going to clinical trials.

In conclusion, the current study is the first to report the advantageous outcomes of coating rhSLPI on titanium to promote the effects of osteogenic adhesion, proliferation, and differentiation. Thus, the outcome of this study can be applied to further investigations through an in vivo model to evidence the clinical potential of the technique.

## Materials and Methods

### Chemical and reagents

Recombinant human SLPI (rhSLPI) was purchased from Sino Biology Inc. (Beijing, China), Dulbecco's Modified Eagle Medium: Nutrient Mixture F12 (DMEM F12) (Gibco BRL; Life Technologies Inc. New York, USA). Dexamethasone, L-2-ascorbic acid, β-glycerophosphate, and Ham’s F-12 media were purchased from Sigma-Aldrich (USA). The phosphate buffer saline (PBS) tablet and the 3-(4,5-dimethylthiazol-2-yl)-2,5-diphenyltetrazolium bromide (MTT) were purchased (Amresco, Ohio, USA). The other chemicals were obtained from Sigma. Total RNA was isolated by PureLink™ RNA Mini Kit (Invitrogen), cDNA was synthesized by Tetro™ cDNA synthesis kit, and real-time PCR was performed by SensiFAST™ SYBR^®^ Hi-ROX kit (Bioline, Tennessee, USA). The human SLPI ELISA kit, Alizarin Red S staining kit, and histochemical stains were bought (Abcam, UK).

Pure, Grade 5 titanium (Ti) discs were customized to 0.5 mm in thickness and 5.0 mm in diameter to fit in a 96-well plate. Similarly, the dimensions of Ti discs were also customized to be 0.5 mm in thickness and 12.0 mm in diameter to fit in a 24-well plate. The Ti discs were sterilized and autoclaved before use.

### Cell type and cell culture

Human fetal osteoblast 1.19 (hFOB 1.19) cells were obtained from the American Type Culture Collection (ATCC CRL-11372TM). Cells were cultured with a 1:1 mixture of Ham’s F12 medium and Dulbecco’s Modified Eagle’s medium containing 10% (v/v) fetal bovine serum (FBS) and 1% penicillin: streptomycin without phenol red.

### Optimization of osteoblast adhesion

The osteoblast cell adhesion was optimized by seeding osteoblasts on cell culture wells for different durations. The number of adherent cells was indirectly assessed using a colorimetric cell–cell adhesion experiment with MTT^19^. Firstly, 2.5 × 10^5^ cells/ml cells were seeded in a 96-well plate and cultured at 37 °C with 5% CO_2_ and 95% air. At each time period (10, 20, 30, and 60 min), the media was removed and renewed. The cells were then cultured for 1 h at 37 °C with 5% CO_2_ and 95% air. After incubation, the number of adherent cells was indirectly determined using the MTT assay.

### Coating titanium surface with rhSLPI

The 5.0 mm-Ti discs were placed into wells of the 96-well plate. Several concentrations of rhSLPI were then diluted in PBS to final concentrations of 0.1, 1, 10, and 100 µg/ml and were added into each well on the Ti surface. The 96-well plate was then incubated at 37 °C with 5% CO_2_ and 95% air overnight. The rhSLPI solution was aspirated the following day and was used for osteoblast adhesion assay.

### Cell viability assay

The hFOB 1.19 cells were seeded on the Ti discs (5 × 10^5^ cells/ml) coated with rhSLPI at concentrations of 0, 10, or 100 µg/ml and were incubated for 24 h. At the end of cell experimental procedures, the culture medium was removed, 0.5 mg/ml MTT solution was applied, and incubation at 37 °C with 5% CO_2_ and 95% air was followed for 2 h in a dark environment. After incubation, the MTT solution was removed, and DMSO solution was mixed to dissolve formazan crystals. Spectrophotometry was performed at 570 nm; the absorbance was measured (O.D. 570 nm).

### Surface characterization

The surface characterization was performed by determining the surface of coating by scanning electron microscopy (SEM) at 2000× magnifications using the Ultrahigh Resolution Field Emission SEM—JSM-IT800 (JEOL, Tokyo, Japan). The surface roughness of the Ti with and without the presence of the rhSLPI coating was determined by Atomic Force Microscopy (AFM) measurements XE 70 model (Park System, South Korea).

The hydrophobic properties of the titanium surface were assessed using the contact angle hysteresis method. A volume of 5 µl of deionized water was applied onto the titanium (Ti) surface under two conditions: with and without the presence of the rhSLPI coating. The captured images in JPEG format, obtained using the camera program, were subsequently imported into ImageJ for analysis. The angles of interest were then identified utilizing the angle tool feature provided by the software. The angles were determined by computing the meaning of three observations obtained from distinct places.

### Determination of rhSLPI concentration on coated-Ti surface by enzyme-linked immunosorbent assay (ELISA)

The concentration of rhSLPI on the coated-Ti surface was measured by a single-wash sandwich ELISA kit ab263890 from Abcam (Cambridge, UK) according to the manufacturer’s instructions. After coating with rhSLPI on Ti for 24 h, the Ti discs were washed three times with 300 µL of PBS with vigorous shaking for 5 min, followed by air-drying. The Ti discs were carefully removed from the wells and transferred to wells in a new 96-well plate. After that, 50 µL of antibody cocktail was added and incubated at room temperature for 1 h. At the end of the incubation period, the reaction was aspirated, and Ti discs were washed three times with 350 µL of wash buffer. The wash buffer was then removed and 100 µL of TMB Development Solution was added to each well for 10 min. Then, 100 µL of stop solution was added and the absorbance was measured at O.D. 450 nm.

### Determination of cellular morphology by scanning electron microscopy (SEM)

The hFOB 1.19 cells were seeded on the Ti discs (5 × 10^5^ cells/ml) coated with rhSLPI at concentrations of 0, 10, or 100 µg/ml and were incubated for 20 or 60 min. During the post-incubation period, the culture medium was aspirated, and 2.5% (v/v) glutaraldehyde was added and incubated at room temperature for 30 min. The Ti discs were carefully removed from the well plate and placed in the oven to dry overnight. Top-view and lateral-view images were taken at 3000× and 5000× magnifications using the JCM-7000 Benchtop Scanning Electron Microscope (SEM) (JEOL, Tokyo, Japan).

### Atomic force microscopy (AFM)

The hFOB 1.19 cells were seeded on the Ti discs (5 × 10^5^ cells/ml) coated with rhSLPI at concentrations of 0, 10, or 100 µg/ml and were incubated for 20 min before fixing the cells with 4% (v/v) paraformaldehyde (PFA) for 10 min^25^. Atomic Force Microscopy (AFM) measurements were performed with an XE 70 model (Park System, South Korea) in contact mode with NCS36 cantilevers with a tip curvature radius below 10 nm, a scan rate of 0.5 Hz, and a scan area of 40 × 40 µm^2^ to determine the surface morphology and Young’s modulus.

### Determination of actin cytoskeleton organization

Following cell adhesion on Ti discs for 20 min, the actin cytoskeletal organization was observed by staining cells with TRITC-conjugated Phalloidin^26^. Cells were briefly washed twice with PBS and fixed for 30 min with 4% formaldehyde before being permeabilized with 0.5% (v/v) Triton-X 100 for 20 min at room temperature. After that, cells were incubated in a dark moist chamber for 40 min with TRITC-conjugated Phalloidin (Sigma, St. Louis, MO, USA) before being washed twice with PBS and viewed under a fluorescent microscope (EVOS M5000 Imaging System, Thermo Fisher Scientific, USA).

For confocal imaging, the hFOB 1.19 cells were stained with TRITC-conjugated Phalloidin and then washed with PBS, cells were counterstained with DAPI (Sigma, St. Louis, MO, USA) for 5 min. The fine details on the organization of the actin cytoskeleton were observed by confocal microscope ZEISS LSM 900 with Airyscan 2 (Carl-Zeiss, Germany).

### Cell proliferation (MTT) assay

The Ti discs were coated with 0, 10, or 100 µg/ml of rhSLPI overnight. rhSLPI was discarded, 200 µl of 1 × 10^4^ cells/ml hFOB 1.19 cells suspension were seeded, and cells were incubated for 3 and 5 days at 95% air and 5% CO_2_ at 37 °C. After the number of designated days of incubation, the supernatant was aspirated, and the cell proliferation was determined by using MTT cell survival assay.

### Osteogenic differentiation

The hFOB 1.19 cells were seeded on the 12.0 mm-Ti discs in 24-well plates at a density of 15,000 cells/well in DMEM/F12 supplemented with 10% FBS for 24 h. Then, the culture medium was discarded and replaced by an osteogenic induction medium containing 0.01 μM dexamethasone, 50 μg/ml ascorbic acid, and 10 mM sodium β-glycerophosphate^24^. The medium was discarded and replaced with a new osteogenic induction medium every three days. After seven days of induction, the osteogenic differentiation potential would be examined by assessing Alizarin Red S (ARS) staining. Relative expressions of osteoblast differentiation gene markers, including alkaline phosphatase (ALP), osteocalcin (OCN), runt-related transcription factor 2 (Runx2), and collagen type I alpha 1 (COL1A1) were determined by reverse transcription-quantitative polymerase chain reaction (RT-qPCR).

### Gene expression by reverse transcription-quantitative polymerase chain reaction (RT-qPCR)

Total RNA was extracted from cells, cDNA was synthesized, and quantitative reverse-transcription PCR was performed as described previously^27^.

In determining gene expression of osteoblast adhesion molecules, a density of 15,000 cells/well of hFOB 1.19 cells were seeded on Ti-discs in 24-well plates in DMEM/F12 supplemented with 10% FBS for 7 days. Extracted RNA was converted to cDNA using oligo dT primers. Then, quantitative polymerase chain reaction (qPCR) was conducted for integrins α1, α2, α5, and β1 by utilizing synthesized primers listed in Table 1^28^.

**Table 1:** Primers used in qPCR to determine gene expression of osteoblast adhesion molecules.

PCR primers	Sequences	*Tm* (°C)	Annealing temperature
H-GAPDH-F	GCTCTCCAGAACATCATCC	52.70	48.00
H-GAPDH-R	TGCTTCACCACCTTCTTG	52.50	
H-ITGα1-F	CACTCGTAAATGCCAAGAAAAG	52.40	47.00
H-ITGα1-R	TAGAACCCAACACAAAGATGC	53.10	
H-ITGα2-F	ACTGTTCAAGGAGGAGAC	51.00	46.00
H-ITGα2-R	GGTCAAAGGCTTGTTTAGG	51.20	
H-ITGα5-F	ATCTGTGTGCCTGACCTG	54.60	50.00
H-ITGα5-R	AAGTTCCCTGGGTGTCTG	54.30	
H-ITGβ1-F	TCCTCCTCATTTCATTCATC	48.90	45.00
H-ITGβ1-R	ATTACTCAGATCCAACCAC	49.40	

Gene expressions of osteoblast differentiation gene markers, including alkaline phosphatase (ALP), osteocalcin (OCN), runt-related transcription factor 2 (Runx2), and collagen type I alpha 1 (COL1A1) were determined by reverse transcription-quantitative polymerase chain reaction (RT-qPCR). The sequences of primers are listed in Table 2^29^.

**Table 2:** Primers used in qPCR to determine gene expression of osteoblast differentiation markers.

PCR primers	Sequences	Tm (°C)	Annealing temperature
H-GAPDH-F	GCTCTCCAGAACATCATCC	52.70	48.00
H-GAPDH-R	TGCTTCACCACCTTCTTG	52.50	
H-ALPL-F	CCAAGGACGCTGGGAAATCT	58.00	53.00
H-ALPL-R	TATGCATGAGCTGGTAGGCG	58.00	
H-OCN-F	CTCACACTCCTCGCCCTAT	57.00	52.00
H-OCN-R	GGTCTCTTCACTACCTCGCTG	58.00	
H-RUNX2-F	GCGCATTCCTCATCCCAGTA	58.00	53.00
H-RUNX2-R	GGCTCAGGTAGGAGGGGTAA	59.00	
H-COL1A1-F	TCTAGACATGTTCAGCTTTGTGGAC	57.00	52.00
H-COL1A1-R	TCTGTACGCAGGTGATTGGTG	58.00	

Relative gene expression levels were analyzed and calculated by normalizing the differences in cycle threshold number (CT) with an internal control reference gene, glyceraldehyde-3-phosphate dehydrogenase (GAPDH)^25^. Calculations of relative gene expression levels were determined by following the ∆∆CT method by Bio-Rad^®^ CFX Manager™ 3.1 software. Levels of gene expression were shown as the relative expression (fold change) by presenting the ratio of gene expression in different conditions relative to the GAPDH control reference gene.

### Determination of mineralization

The mineralization of differentiated hFOB 1.19 cells was examined through Alizarin Red S Staining. The protocol was conducted by following the instruction manual from Alizarin Red S (Abcam, UK). The hFOB 1.19 cells were seeded on the Ti discs with and without rhSLPI (10 µg/ml) coating and cultured in the presence and absence of osteogenic induction medium for 14 days. At the end of culture period, cells were initially fixed with 4% paraformaldehyde, washed with PBS, and stained with 40 mM Alizarin Red S. After staining, the Ti discs were carefully removed from the wells of the 96-well plate and placed on a white background surface. Photos of the Ti discs were then taken. The calcium concentration in culture medium was measured by automate biochemical analyzer Roche Cobas C111.

### Determination of alkaline phosphatase activity

In assessing the effects of rhSLPI on hFOB 1.19 cells’ differentiation, the activity of alkaline phosphatase was qualitatively determined through alkaline phosphatase staining (Alkaline Phosphatase Staining Kit (Purple), Abcam, UK). The hFOB 1.19 cells were seeded on the Ti discs with and without rhSLPI (10 µg/ml) coating and cultured in the presence and absence of osteogenic induction medium for 14 days. At the end of culture period, the culture medium was removed, cells were washed with 1× PBS containing 0.05% Tween-20 (PBS-T) and were fixed using the Fixing Solution, followed by washing with PBS-T. In a dark box, cells were stained by the AP Staining Solution for 30 min at room temperature. The AP Staining Solution was later removed and washed with 1X PBS. Photos of the Ti discs were then taken. The ALP activity in culture medium was measured by automate biochemical analyzer Roche Cobas C111.

### Statistical analysis

Statistical tests were conducted using commercially available software (GraphPad Prism). All values were expressed as mean ± S.D. Comparisons between Ti and rhSLPI + Ti groups were performed by the unpaired t-test when comparisons in other experiments were assessed using ANOVA to test for significance, followed by, when appropriate, the Tukey–Kramer test. A *p*-value of less than 0.05 was considered statistically significant.

## Data Availability

The datasets generated and/or analyzed during the current study are available in the OSFHOME repository, DOI 10.17605/OSF.IO/NRK5F.
